# Abrogation of CC chemokine receptor 9 ameliorates collagen-induced arthritis of mice

**DOI:** 10.1186/s13075-014-0445-9

**Published:** 2014-09-24

**Authors:** Waka Yokoyama, Hitoshi Kohsaka, Kayoko Kaneko, Matthew Walters, Aiko Takayasu, Shin Fukuda, Chie Miyabe, Yoshishige Miyabe, Paul E Love, Nobuhiro Nakamoto, Takanori Kanai, Kaori Watanabe-Imai, Trevor T Charvat, Mark ET Penfold, Juan Jaen, Thomas J Schall, Masayoshi Harigai, Nobuyuki Miyasaka, Toshihiro Nanki

**Affiliations:** Department of Rheumatology, Graduate School of Medical and Dental Sciences, Tokyo Medical and Dental University, 1-5-45, Yusima, Bunkyo-ku, Tokyo 110-8519 Japan; ChemoCentryx, Inc, 850 Maude Avenue, Mountain View, CA 94043 USA; Department of Dermatology, Tokyo Medical University, 6-1-1 Shinjuku, Shinjuku-ku, Tokyo 160-8402 Japan; National Institute of Child Health and Human Development, National Institutes of Health, 9000 Rockville Pike, Bethesda, MD 20892 USA; Division of Gastroenterology and Hepatology, Department of Internal Medicine, Keio University School of Medicine, 35 Shinanomachi, Shinjuku-ku, Tokyo 160-8582 Japan; Department of Clinical Research Medicine, Teikyo University, 2-11-1 Kaga, Itabashi-ku, Tokyo 173-8605 Japan

## Abstract

**Introduction:**

Biological drugs are effective in patients with rheumatoid arthritis (RA), but increase severe infections. The CC chemokine receptor (CCR) 9 antagonist was effective for Crohn’s disease without critical adverse effects including infections in clinical trials. The present study was carried out to explore the pathogenic roles of chemokine (C-C motif) ligand (CCL) 25 and its receptor, CCR9, in autoimmune arthritis and to study if the CCR9 antagonist could be a new treatment for RA.

**Methods:**

CCL25 and CCR9 expression was examined with immunohistochemistry and Western blotting. Concentration of interleukin (IL)-6, matrix metalloproteinase (MMP)-3 and tumor necrosis factor (TNF)-α was measured with enzyme-linked immunosorbent assays. Effects of abrogating CCR9 on collagen-induced arthritis (CIA) was evaluated using CCR9-deficient mice or the CCR9 antagonist, CCX8037. Fluorescence labeled-CD11b^+^ splenocytes from CIA mice were transferred to recipient CIA mice and those infiltrating into the synovial tissues of the recipient mice were counted.

**Results:**

CCL25 and CCR9 proteins were found in the RA synovial tissues. CCR9 was expressed on macrophages, fibroblast-like synoviocytes (FLS) and dendritic cells in the synovial tissues. Stimulation with CCL25 increased IL-6 and MMP-3 production from RA FLS, and IL-6 and TNF-α production from peripheral blood monocytes. CIA was suppressed in CCR9-deficient mice. CCX8037 also inhibited CIA and the migration of transferred CD11b^+^ splenocytes into the synovial tissues.

**Conclusions:**

The interaction between CCL25 and CCR9 may play important roles in cell infiltration into the RA synovial tissues and inflammatory mediator production. Blocking CCL25 or CCR9 may represent a novel safe therapy for RA.

## Introduction

Rheumatoid arthritis (RA) is characterized by persistent and erosive arthritis in multiple joints. The accumulation of a large number of T cells and macrophages [1-3], proliferation of fibroblast-like synoviocytes (FLS), production of inflammatory mediators and activation of osteoclasts are revealed in the affected joints and lead to destruction of the joints with pain and daily disability [4-8]. Biological drugs, such as tumor necrosis factor (TNF) blockers and interleukin (IL)-6 receptor antagonists, are effective in patients with RA [9-11]. Since the risk of severe infections is increased by biological drugs [12-14], safer therapies for RA should be developed.

As a new treatment, anti-chemokine therapy has been intensively studied for inflammatory diseases. Chemokines are a family of small secreted molecules that induce directed chemotaxis of responding cells and activation of inflammatory cells [15-17]. According to the results of a large phase II study, the CC chemokine receptor (CCR) 9 antagonist, CCX282-B was effective for Crohn’s disease without critical adverse effects [18,19]. Especially, this treatment did not increase the risk of infections for 12 months. CCR9, a unique receptor for chemokine (C-C motif) ligand (CCL) 25, is expressed on lymphocytes of intestinal lamina propria and intraepithelial and dendritic cells (DCs) in the small intestine and thymocytes [20,21]. CCL25 is expressed by the follicle-associated epithelium of Peyer’s patches, the crypts of Lieberkühn in the small intestine and the thymus [21,22]. Physiologically, the interaction between CCL25 and CCR9 contribute to the T cell and DC migration into the small intestine and movement of T cells in the thymus.

It was reported that CCR9 expression on cell surface of peripheral blood monocytes from RA patients was higher than that from healthy donors [23]. CCR9 and CCL25 were expressed on macrophages in the RA synovial tissues [23]. These data suggest that interaction of CCL25 and CCR9 may contribute to the inflammatory cell migration into the RA synovial tissues. Although blockade of CCL25 and CCR9 interaction might also be applicable to RA, the pathogenic roles of these molecules in RA have been little known.

In this study, we examined the stimulatory effects of CCL25 on FLS and monocytes and effects of the abrogation of CCR9 on a murine model of RA.

## Methods

### Specimens

Synovial tissue samples were obtained from eleven RA patients who fulfilled the American College of Rheumatology classification criteria for RA [24] and seven patients with osteoarthritis (OA) who underwent total knee joint replacement. Nine RA patients were positive for rheumatoid factor (81%) and ten were positive for anti-citrullinated protein antibodies (91%). All subjects provided written informed consent. The experimental protocols were approved by the Ethics Committee of Tokyo Medical and Dental University.

### Immunodetection

Mouse anti-CCR9 (248621: R&D Systems, Minneapolis, MN, USA), CCL25 (52513: R&D Systems), or β-actin (AC-15: Sigma-Aldrich, St Louis, MO, USA) monoclonal antibody (mAb) was used as a primary antibody for Western blotting [25]. Immunohistochemistry was conducted as described previously [25]. Frozen sections fixed with ice-cold acetone was blocked with Tris-buffered saline, 2% goat serum, 1% bovine serum albumin, 0.1% Triton X-100, and 0.05% Tween-20. Mouse anti-CCR9, CCL25 mAb (10 μg/ml: R&D Systems) or isotype control was used as a primary antibody. Alexa Fluor™ 546-conjugated goat anti-mouse IgG2a or IgG2b Ab (4 μg/ml: Invitrogen, Carlsbad, CA, USA) was used as a secondary antibody. For double immunohistochemistry, the sections were also stained with mouse anti-CD68 (10 μg/ml: KP1; Dako, Glostrup, Denmark), cadherin-11 (1 μg/ml: 16A; Acris Antibodies, Hiddenhausen, Germany), or dendritic cell lysosome-associated membrane glycoprotein (DC-LAMP) (10 μg/ml: 104.G4; Immunotec Inc., Quebec, Canada) mAb. They were then incubated with 4 μg/ml Alexa Fluor™ 488-conjugated goat anti-mouse IgG1 (Invitrogen). A nuclear stain was performed with 4’ , 6-diamidino-2-phenylindole. To determine the percentages of CCR9-expressed cells, the number of CCR9-positive cells in CD68-, cadherin-11-, or DC-LAMP-positive cells was counted in three randomly selected fields examined at x200 magnification under fluorescence microscope.

### Cell culture

FLS was established from the RA and OA synovial tissues and used for experiments after five passages [26]. The cells did not express CD14 or human leukocyte antigen class II, suggesting that macrophages and DCs were not contained in the FLS [26]. Human peripheral blood CD14^+^ monocytes from healthy donors were purified by magnetic-activated cell sorting microbeads coupled with mAb and magnetic cell separation columns (Miltenyi Biotec, Bergisch Gladbach, Germany). The purity of CD14^+^ monocytes was more than 95%. CCR9 expression of RA FLS and purified human peripheral blood monocytes was evaluated with phycoerythrin-conjugated anti-CCR9 mAb (112509: R&D Systems) or the isotype control staining using an Accuri C6 flow cytometer (Accuri Cytometers, BD Biosciences, San Jose, CA, USA). RA FLS were cultured with 10% fetal calf serum (FCS) for 48 hours with or without recombinant human CCL25 (R&D Systems). Purified human peripheral blood monocytes were cultured with 10% FCS for 24 hours with or without CCL25. Concentrations of IL-6, matrix metalloproteinase (MMP)-3 and TNF-α in the culture supernatants were measured with enzyme-linked immunosorbent assay (ELISA) kits (DuoSet: R&D Systems).

### Induction and treatment of collagen-induced arthritis (CIA)

To induce CIA, 10-week-old CCR9-deficient mice [27] and wild-type (WT) mice with a C57BL/6 J background were treated with chicken type II collagen (CII; Sigma-Aldrich) [28]. Eight-week-old DBA/1 J mice were treated with bovine CII (Collagen Research Center, Kiel, Germany) [29]. Disease severity was evaluated with the clinical arthritis score, incidence of arthritis, pathological score [29] and radiological score. Bone destruction was evaluated with bone erosion of the bilateral foot joints as follows: 0 = not obvious erosion, 1 = one erosion, 2 = two erosions, 3 = more than three erosions, and if a bone deformity was seen in the foot joint, one point was added (to a maximum of eight points).

DBA/1 J mice with CIA were injected subcutaneously with selective CCR9 antagonist, CCX8037 [30] or vehicle alone. Migration of CD11b^+^ splenocytes into synovial tissues of CIA mice was evaluated as described previously [31,32]. CD11b-positive splenocytes from CIA mice were purified using magnetic-activated cell sorting microbeads coupled with mAb and magnetic cell separation columns (Miltenyi Biotec). The purified CD11b cells were labeled with CellTracker™ Orange 5-(and-6)-(((4-chloromethyl) benzoyl) amino) tetramethylrhodamine (CMTMR: Molecular Probes, Eugene, OR, USA) according to the protocol supplied by the manufacturer. The CMTMR-labeled 1 × 10^7^ cells were intravenously injected into the tail vein of CIA mice at day 9. The recipient mice were treated with CCX8037 (10 mg/kg in 1% hydroxyprophyl methylcellulose) or vehicle 24 hours, 12 hours, and 30 minutes before the transfer, and 12 hours after the transfer. After 24 hours, ankle joints were harvested, embedded in glycol methacrylate, and sagittal 3-μm-thick microtome sections were prepared. The numbers of CMTMR-labeled cells that migrated into the synovium between tibiotalar and tarsometatarsal joints were counted under fluorescent microscopy. The experiment protocols were approved by the Institutional Animal Care and Use Committee of Tokyo Medical and Dental University.

### Statistical analysis

To compare three or more groups, including results of concentration of inflammatory mediators, area under the curve (AUC) by arthritis score, histological and radiological scores by therapeutical treatment, a one-way analysis of variance (ANOVA) test was used. To compare arthritis score, a two-way ANOVA test was used. AUC by arthritis score, histological score and radiographic score by prophylactic treatment or experiments using CCR9-deficient mice, and number of migrated CD11b^+^ splenocytes, Student’s *t* test was also applied. A *P* value less than 0.05 was considered statistically significant.

## Results

### Expression of CCR9 and CCL25 in the RA synovial tissues

**Figure 1:**
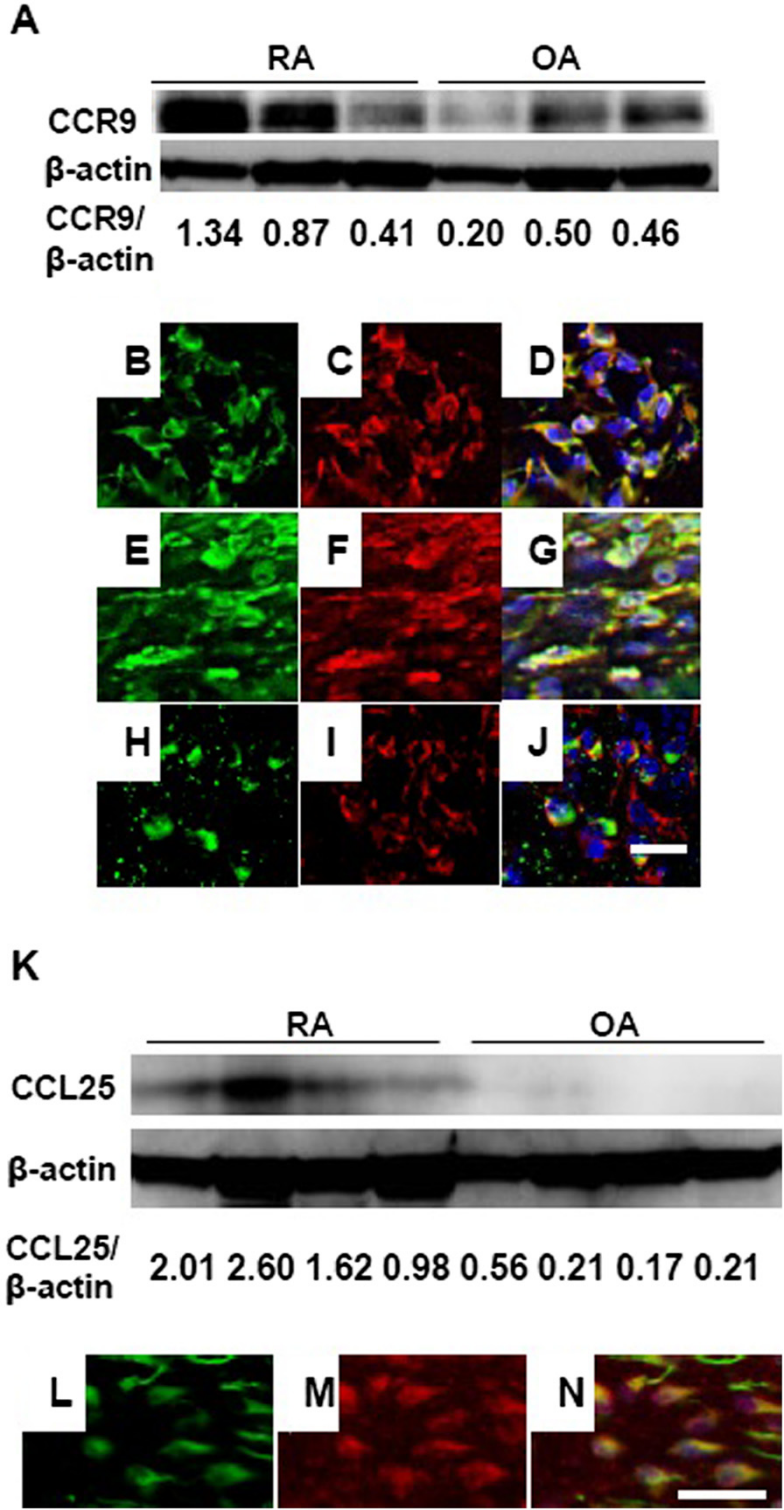
**CCR9 and CCL25 expression in the RA synovial tissues. (A)** Western blots of CCR9 protein expression in three RA and three OA synovial tissues. Ratio of CCR9/β-actin protein level is indicated below. **(B-J)** Sections of RA synovial tissue were double-stained with CCR9 and CD68, cadherin-11 and DC-LAMP (B, CD68; C, CCR9; D, merged B with C; E, cadherin-11; F, CCR9; G, merged E with F; H, DC-LAMP; I, CCR9; J, merged H with I). Bar, 20 μm. **(K)** Western blots of CCL25 protein expression in four RA and four OA synovial tissues. Ratio of CCL25/β-actin protein level is indicated below. **(L-N)** Sections of RA synovial tissue were double-stained with CCL25 and CD68 (L, CD68; M, CCL25; N, merged L with M). Bars, 20 μm. CCL, chemokine (C-C motif) ligand; CCR, CC chemokine receptor; DC-LAMP, dendritic cell lysosome-associated membrane glycoprotein; OA, osteoarthritis; RA, rheumatoid arthritis.

CCR9 expression in RA and OA synovial tissues was evaluated with Western blotting. The CCR9 protein was found more in the RA synovial tissues than in the OA synovial tissues (Figure 1A). Immunohistochemical analyses of the RA synovial tissues revealed that most CD68^+^ macrophages expressed CCR9 (Figure 1B-D), which is consistent with previous reports [23]. To examine the proportion of CCR9-expressed cells, the number of CCR9-positive cells in 218.83 ± 24.11 CD68^+^ cells (n =3), 206.33 ± 23.08 cadherin-11^+^ cells (n =3) and 11.33 ± 0.53 DC-LAMP^+^ cells (n =3) was counted. The percentage of CCR9-positive cells in CD68^+^ macrophages was 76.1 ± 5.3%. In addition, cadherin-11^+^ FLS and DC-LAMP^+^ DCs also expressed CCR9 (67.1 ± 8.3%, 11.4 ± 15.2%, respectively) (Figure 1E- J).

The CCL25 expression in RA and OA synovial tissues was analyzed with Western blotting. CCL25 was expressed more in the RA synovial tissues than in the OA synovial tissues (Figure 1K). Double immunohistochemistry revealed that CD68^+^ macrophages expressed CCL25 in the RA synovial tissues (Figure 1L-N), which is consistent with previous reports [23].

### Stimulatory effects of CCL25 on RA FLS and human peripheral blood monocytes

**Figure 2:**
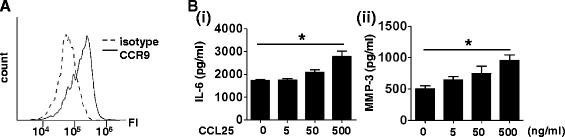
**Effects of CCL25 on cultured RA FLS. (A)** CCR9 expression on *in vitro* cultured RA FLS was determined by flow cytometry. Horizontal line indicates fluorescence intensity (FI). **(B)** RA FLS were cultured for 48 hours with various concentrations of CCL25. Concentrations of IL-6 (i) and MMP-3 (ii) in the culture supernatant were measured with ELISA. Values are the mean ± SEM of three independent experiments. *P <0.05. CCL, chemokine (C-C motif) ligand; CCR, CC chemokine receptor; ELISA, enzyme-linked immunosorbent assay; FLS, fibroblast-like synoviocytes; IL, interleukin; MMP, metalloproteinase; RA, rheumatoid arthritis; SEM, standard error of the mean.

Since FLS expressed CCR9 in the RA synovial tissues, we examined the stimulatory effects of CCL25 on the cultured RA FLS, which also expressed CCR9 (Figure 2A; mean fluorescence intensity (MFI) for CCR9: 157,963 ± 37,811, MFI for isotype control: 70,914 ± 3,163 (mean ± standard error of the mean (SEM)), n =3). We cultured RA FLS for 48 hours with various concentrations of CCL25. Stimulation with CCL25 increased IL-6 and MMP-3 levels in the culture supernatants in a dose-dependent manner (Figure 2B). Production of TNF-α was not detected by unstimulated or CCL25-stimulated RA FLS. We next examined the effect of CCL25 on IL-6 and MMP-3 production by OA FLS. IL-6 and MMP-3 were also secreted from unstimulated OA FLS, although the levels were lower than those from RA FLS (IL-6; RA: 1,393 ± 493 mg/dl (n =3), OA: 171 ± 83 mg/dl (n =2), *P* <0.05, MMP-3; RA: 2135 ± 644 mg/dl, OA: 296 ± 50 mg/dl, *P* <0.05). IL-6 secretion from OA FLS was slightly increased by CCL25, although it was not statistically significant (CCL25 5 ng/ml: 185 ± 93 mg/dl, 50 ng/ml: 206 ± 120 mg/dl, 500 ng/ml: 243 ± 123 mg/dl). Production of MMP-3 from OA FLS was not significantly altered by CCL25 (CCL25 5 ng/ml: 324 ± 115 mg/dl, 50 ng/ml: 324 ± 65 mg/dl, 500 ng/ml: 312 ± 112 mg/dl).

**Figure 3:**
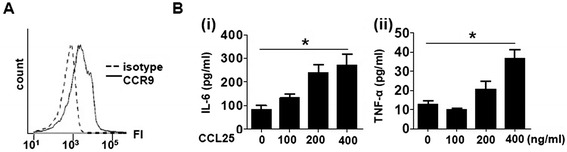
**Effects of CCL25 on human peripheral blood monocytes. (A)** CCR9 expression on peripheral blood CD14^+^ monocytes was analyzed with flow cytometry. **(B)** Monocytes were cultured for 24 hours with various concentrations of CCL25. Concentrations of IL-6 (i) and TNF-α (ii) in the culture supernatant were measured with ELISA. Values are the mean ± SEM of three independent experiments. *P <0.05. CCL, chemokine (C-C motif) ligand; CCR, CC chemokine receptor; ELISA, enzyme-linked immunosorbent assay; IL, interleukin; SEM, standard error of the mean; TNF, tumor necrosis factor.

Instead of the synovial macrophages, which were not available on a large scale, we examined the effect of CCL25 on human peripheral blood monocytes. They also expressed CCR9 (Figure 3A; MFI for CCR9: 24,149 ± 13,536, MFI for isotype control: 2,038 ± 1,265, n =3) and were cultured for 24 hours with various concentrations of CCL25. This treatment promoted IL-6 and TNF-α production in a dose-dependent manner (Figure 3B).

### Effects of CCR9 gene deletion on murine CIA

**Figure 4:**
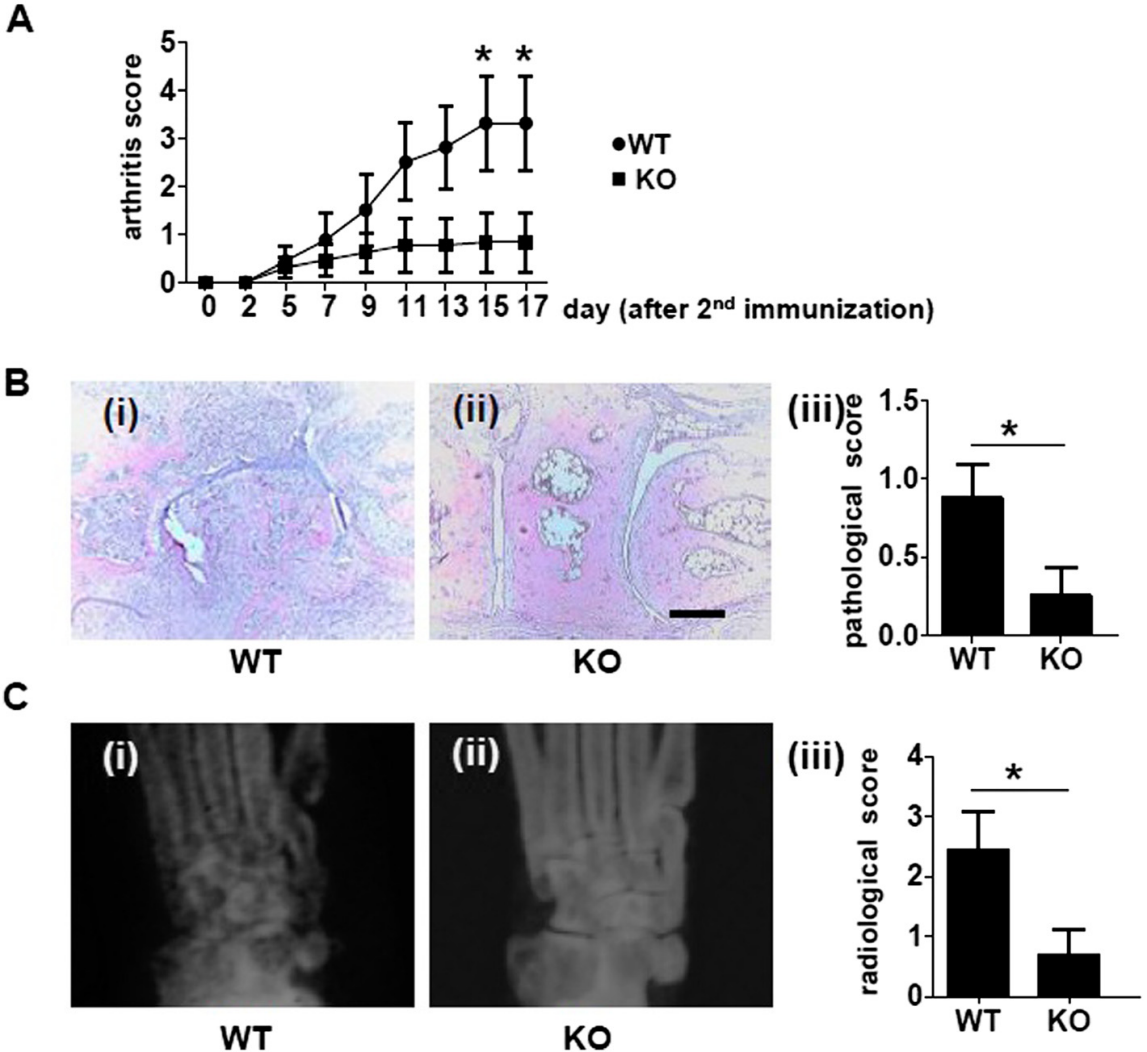
**Suppressed CIA in CCR9-deficient mice. (A-C)** CCR9-deficient mice (knockout (KO)) (n =13) and WT mice (n =16) were immunized with chicken CII on day −21 and 0. Day 0 means the day of the second immunization. Disease severity was recorded as the clinical arthritis score until day 18 (A). Ankle joints on day 18 from KO (B(i)) and WT mice (B(ii)) were stained with hematoxylin and eosin. Bar, 300 μm. Inflammatory cell infiltration in the right ankle joint was scored with a pathological score (B(iii)). Representative radiographs of the ankle joints of KO (C(i)) and WT mice (C(ii)). Bone erosion in the bilateral ankle joints was scored with radiological score (C(iii)). Representative photomicrographs are shown. Values are the mean ± SEM of each group. *P <0.05 versus WT. CCR, CC chemokine receptor; CIA, collagen-induced arthritis; CII, type II collagen; SEM, standard error of the mean; WT, wild-type.

The above data prompted us to investigate the effect of the abrogation of CCR9 on murine CIA. We analyzed the development of CIA in CCR9-deficient mice. The clinical arthritis scores in the CCR9-deficient mice were significantly lower than those in the WT (Figure 4A). AUC by the arthritis score was calculated. The AUC tended to be smaller in CCR9-deficient mice compared to WT mice, although the difference was not statistical significant (WT: 13.40 ± 17.75, CCR9-deficient: 4.08 ± 10.34). Histological and radiographic examinations revealed that mononuclear cell infiltration and bone destruction were inhibited in the CCR9-deficient mice (Figure 4B and C).

### Effects of the CCR9 antagonist on murine CIA

**Figure 5:**
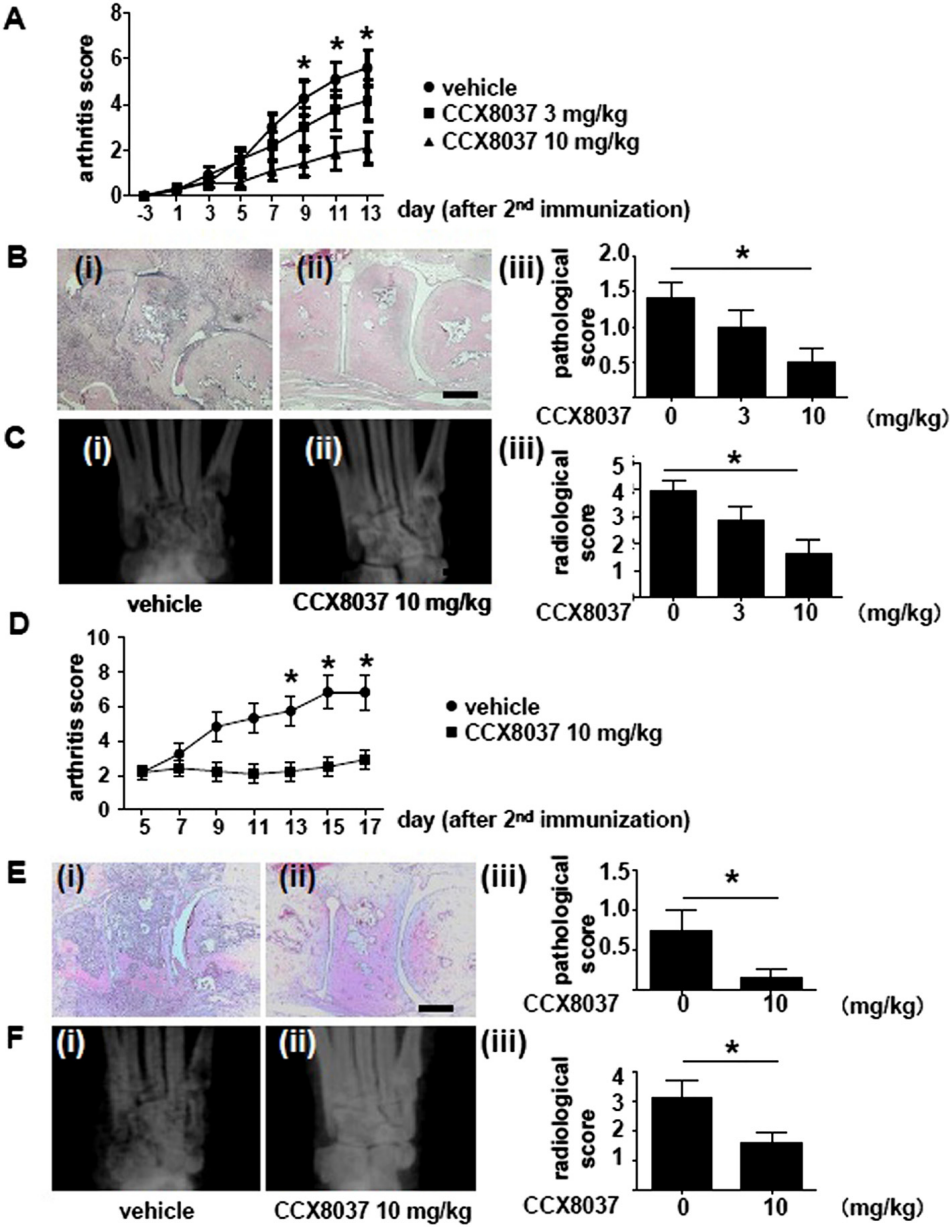
**Effects of CCX8037 on CIA mice. (A-C)** CCX8037 (3 mg/kg or 10 mg/kg) or vehicle (all n =12) was injected subcutaneously twice daily from day −7 to day 14. Disease severity was recorded as the clinical arthritis score until day 14 (A). Representative photographs showing hematoxylin and eosin staining of the ankle joints from mice with CIA treated with vehicle (B(i)) or CCX8037 (10 mg/kg) (B(ii)). Bar, 300 μm. Inflammatory cell infiltration in the right ankle joint was scored with a pathological score (B(iii)). Representative radiographs of the ankle joints of CIA mice treated with vehicle (C(i)) or CCX8037 (10 mg/kg) (C(ii)). Bone erosion in the bilateral ankle joints was scored with radiological score (C(iii)). Values are the mean ± SEM of each group. *P <0.05, CCX8037 (10 mg kg) versus vehicle. **(D-F)** To investigate the therapeutic effects of CCX8037, at day 5 after the second immunization, mice were divided into two groups with equal average arthritis score. CCX8037 (10 mg/kg) or vehicle (all n =12) was injected subcutaneously twice daily from day 5 to day 18. Disease severity was recorded as the clinical arthritis score (D). Representative hematoxylin and eosin staining of the ankle joints of CIA mice treated with vehicle (E(i)) or CCX8037 (10 mg/kg) (E(ii)). Bar, 300 μm. Pathological score (E(iii)). Representative radiographs of the ankle joints of CIA mice treated with vehicle (F(i)) or CCX8037 (10 mg/kg) (F(ii)). Radiological score (F(iii)). Values are the mean ± SEM of each group. *P <0.05 versus vehicle. CCX8037, CC chemokine receptor (CCR) 9 antagonist; CIA, collagen-induced arthritis; SEM, standard error of the mean.

Next, we investigated the effect of the CCR9 antagonist, CCX8037, on murine CIA. To analyze its effects in a preventive protocol, CCX8037 (3 mg/kg or 10 mg/kg) or vehicle alone was injected subcutaneously twice daily from 7 days prior to the second immunization to 14 days after the immunization. This treatment significantly inhibited clinical arthritis score in a dose-dependent manner (Figure 5A). AUC by the arthritis score was also significantly smaller by CCX8037 (10 mg/kg)-treated mice compared with vehicle-treated mice (vehicle: 17.67 ± 11.78, CCX8037 3 mg/kg: 13.67 ± 11.79 (not significant, vs. vehicle), CCX8037 10 mg/kg: 6.79 ± 9.22 (*P* =0.01, vs. vehicle)). Moreover, CCX8037 significantly inhibited mononuclear cell infiltration and bone destruction (Figure 5B and C).

To investigate the effects of CCX8037 in a therapeutic protocol, we injected CIA mice with CCX8037 10 mg/kg or vehicle alone 5 days after the second immunization for 13 days. CCX8037 significantly inhibited arthritis, mononuclear cell infiltration and bone destruction (Figure 5D-F). AUC by the arthritis score was also significantly smaller in CCX8037-treated mice compared with vehicle-treated mice (vehicle: 30.50 ± 16.46, CCX8037 10 mg/kg: 14.46 ± 10.75 (*P* <0.01, vs. vehicle)).

### The CCR9 antagonist inhibited migration of CD11b^+^ splenocytes

In the RA synovial tissues, most macrophages expressed CCR9, and CCL25 was abundant (Figure 1B-D and 1K). It was reported that CCL25 induced migration of monocytes/macrophages *in vitro* [23,33,34], indicating that the interaction of CCL25 and CCR9 may have an important role in the migration of monocytes into the inflamed synovial tissues.

**Figure 6:**
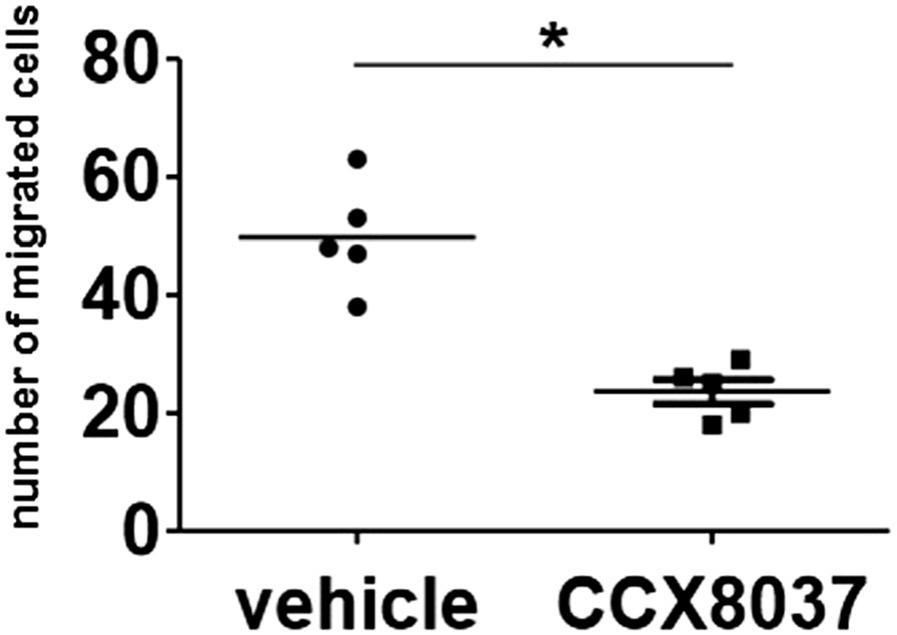
**Effect of CCX8037 on the migration of CD11b** ^**+**^ **splenocytes into the joints.** Cell Tracker Orange CMTMR-labeled CD11b^+^ splenocytes from CIA mice (1 × 10^7^) were adoptively transferred into each recipient CIA mouse on day 9 after the second immunization. The recipient mice were treated with CCX8037 (10 mg/kg in 1% hydroxyprophyl methylcellulose) or vehicle 24 hours, 12 hours, and 30 minutes before the transfer, and 12 hours after the transfer. Twenty-four hours after the transfer, ankle joints were harvested and examined for migrated cells under a fluorescent microscope. The total number of labeled cells in three fields of vision was counted in the synovial tissue between the tibiotalar and transmetatarsal joints (n =5) at x20 magnification. Horizontal bars indicate the mean of each group. *P <0.001 versus vehicle. CCX8037, CC chemokine receptor (CCR) 9 antagonist; CIA, collagen-induced arthritis; CMTMR; 5-(and-6)-(((4-chloromethyl) benzoyl) amino) tetramethylrhodamine.

We then analyzed the effect of CCR9 blockade on inflammatory cell migration *in vivo.* We showed previously that CD11b^+^ macrophages from CIA mice labeled and transferred to the recipient CIA mice were identified in the inflamed synovial tissues of the recipients [31,32]. CD11b^+^ splenocytes express CCR9 [35]. To analyze the effect of CCX8037 on the macrophages migration, recipient mice were treated with CCX8037 or vehicle 24 hours, 12 hours, and 30 minutes before the cell transfer, and 12 hours after the transfer. Twenty-four hours after the transfer, the number of labeled cells in the synovial tissues was counted. Although, this short-term treatment did not alter the arthritis severity of the recipient mice, the treated mice had significantly reduced number of the migrated cells in the synovial tissues in contrast to the vehicle-treated group (Figure 6).

## Discussion

In this study, we showed that the abrogation of CCR9 ameliorated arthritis in a murine model of RA. We also found that the *in vivo* migration of macrophages was suppressed by the administration of CCX8037. In addition, we showed stimulatory effects of CCL25 on the production of inflammatory mediators from RA FLS and human peripheral blood monocytes *in vitro*. The results suggest that CCR9 could be a therapeutic target for RA.

As was reviewed earlier, chemokines are apparent therapeutic targets in RA treatment. Especially, CCR1, CCR2 and CCR5 are abundantly expressed on RA synovial macrophages and the validity of CCR1, CCR2 and CCR5 antagonist for the animal model of arthritis has been studied. CCR1 antagonist was effective in a clinical trial [36], while blockade of CCL2, CCR2 or CCR5 was not [37-39]. In the trial of CCR2 or CCR5 antagonist, no significant reduction in numbers of macrophages in the synovial tissues was observed, suggesting that CCR2 and CCR5 may not play a critical role in the migration of monocytes. In addition, since CCR2 and CCR5 are expressed on regulatory T cells, their blockade might inhibit regulatory T cells that suppressed the disease.

CCR9 was expressed on FLS, macrophages and DCs in the RA synovial tissues. This should be driven by inflammatory cytokines in the synovial tissues, since stimulation with TNF-α increased CCR9 expression on THP monocytic cells [23]. CCR9 was also expressed on *in vitro* cultured RA FLS and peripheral blood monocytes from healthy donors. CCL25 stimulated them to produce inflammatory mediators that are important in the pathogenesis of RA. CCL25 should exacerbate arthritis via these effects in addition to the inflammatory cell recruitment. We could not measure CCL25 concentration in the RA synovial tissue. However, chemokines bind surface proteoglycans [40], and they could be sequestered and presented to target cells at high concentration in the local microenvironment.

*In vivo* macrophage migration was suppressed by CCR9 inhibition, which might have a great impact on inhibition of CIA. It was shown that CD3^+^ T cells and CD20^+^ B cells in the RA synovial tissues did not express CCR9 [23]. In addition, the serum concentration of anti-CII IgG1, IgG2a and IgG2b in the CCX8037-treated group was not lower than the vehicle group both in the preventive and therapeutic treatment experiments (data not shown). The effect of CCX8037 may not depend on the inhibition of T and B cells recruitment into the synovial tissues or reduction of antibody production. It was reported that deficiency of CCR9, as well as CCR1, CCR2 and CCR5, did not attenuate a murine model of serum transfer arthritis [41]. It is believed that innate immune system is important for serum transfer arthritis, while adaptive immune system is important for CIA. Macrophages and neutrophils are essential for serum transfer arthritis [41,42]. Reduction in the number of macrophages in the synovial tissues might not have enough of an impact to suppress the inflammation of arthritis in serum transfer arthritis, while that might suppress CIA. On the other hand, CCR9 was expressed on the DC-LAMP^+^ cell in the synovial tissues, which is a mature DC [43] in the RA synovial tissues. We did not investigate the effect of the CCL25-CCR9 interaction on DCs in this study. Further studies are needed to investigate the regulation of CCR9 expression and effect of CCL25 stimulation on DCs.

Treatment with CCR9 antagonist did not accompany critical infections as an adverse effect in a large phase II study for Crohn’s disease [18,19]. Although the reason is not clear, it might be also the case for treating RA.

## Conclusions

In this manuscript, we showed that CCL25 and CCR9 were expressed in the RA synovial tissue. Stimulation with CCL25 enhanced production of inflammatory mediators from monocytes and RA FLS. Moreover, inhibition of CCR9 reduced arthritis and inflammatory cell migration in mice. Therefore, the interaction between CCL25 and CCR9 may play important roles in cell infiltration into the RA synovial tissues and inflammatory mediator production. CCR9 antagonist may become a novel, safe and effective treatment for RA.

## Abbreviations

ANOVA: analysis of variance
AUC: area under the curve
CCL: chemokine (C-C motif) ligand
CCR: CC chemokine receptor
CIA: collagen-induced arthritis
CII: type II collagen
CMTMR: 5-(and-6)-(((4-chloromethyl)benzoyl)amino)tetramethylrhodamine
DC: dendritic cell, DC-LAMP, dendritic cell lysosome-associated membrane glycoprotein
ELISA: enzyme-linked immunosorbent assays
FCS: fetal calf serum
FI: fluorescence intensity
FLS: fibroblast-like synoviocytes
IL: interleukin
KO: knockout
mAb: monoclonal antibody
MFI: mean fluorescence intensity
MMP: metalloproteinase
OA: osteoarthritis
RA: rheumatoid arthritis
SEM: standard error of the mean
TNF: tumor necrosis factor
WT: wild-type
